# Advances in Mesoporous Silica and Hybrid Nanoparticles for Drug Delivery: Synthesis, Functionalization, and Biomedical Applications

**DOI:** 10.3390/pharmaceutics17121602

**Published:** 2025-12-12

**Authors:** Ahmad Almatroudi

**Affiliations:** Department of Medical Laboratories, College of Applied Medical Sciences, Qassim University, Buraydah 51452, Saudi Arabia; aamtrody@qu.edu.sa

**Keywords:** hybrid nanocarriers, surface functionalization, stimuli-responsive systems, biomedical applications, biocompatibility, clinical translation

## Abstract

Mesoporous silica nanoparticles (MSNs) are among the most adaptable nanocarriers in modern pharmaceutics, characterized by a high surface area, tunable pore size, controllable morphology, and excellent biocompatibility. These qualities enable effective encapsulation, protection, and the delivery of drugs in a specific area and, therefore, MSNs are powerful platforms for the targeted and controlled delivery of drugs and theragnostic agents. Over the past ten years and within the 2021–2025 period, the advancement of MSN design has led to the creation of hybrid nanostructures into polymers, lipids, metals, and biomolecules that have yielded multifunctional carriers with enhanced stability, responsiveness, and biological activities. The current review provides a review of the synthesis methods, surface functionalization techniques, and physicochemical characterization techniques that define the next-generation MSN-based delivery systems. The particular focus is put on stimuli-responsive systems, such as redox, pH, enzyme-activated, and light-activated systems, that enable delivering drugs in a controlled and localized manner. We further provide a summary of the biomedical use of MSNs and their hybrids such as in cancer chemotherapy, gene and nucleic acid delivery, antimicrobial and vaccine delivery, and central nervous system targeting, supported by recent in vivo and in vitro studies. Important evaluations of biocompatibility, immunogenicity, degradation, and biodistribution in vivo are also provided with a focus on safety in addition to the regulatory impediments to clinical translation. The review concludes by saying that there are still limitations such as large-scale reproducibility, long-term toxicity, and standardization by the regulators, and that directions are being taken in the future in the fields of smart programmable nanocarriers, green synthesis, and sustainable manufacture. Overall, mesoporous silica and hybrid nanoparticles represent a breakthrough technology in the nanomedicine sector with potentials that are unrivaled in relation to targeted, controlled, and personalized therapeutic interventions.

## 1. Introduction

Mesoporous silica nanoparticles (MSNs) have elicited a high level of interest among scientists due to their outstanding physicochemical characteristics, which include superior surface area, tunable pore size, thermal stability, and easy accessibility to surface functionalization [1,2,3]. As a result of these properties, MSNs can be considered as possible applicants of a broad spectrum of applications in pharmaceutics, biomedicine, and biotechnology. In the past 10 years, MSNs have been extensively studied as nanocarriers of drug delivery because of their large encapsulation capacity of a wide range of drugs and their ability to deliver the drugs in a controlled and targeted way [4,5]. The silica support is able to provide structural stability and chemical flexibility which allow the mesoporous nature of MSNs to facilitate effective drug loading and the ability to tightly control release kinetics and site-specific delivery of the drug [6,7].

MSNs have been found to have a lot of promise in cancer therapy, antibacterial therapy, gene therapy, and imaging diagnostics in that they are biocompatible and have the ability to be surface-modified with various functional groups, ligands, and polymers [8,9]. Biomolecules such as peptides, antibodies, or aptamers that are bound to the surfaces of MSNs enhance their affinity to a target sick cell or tissue [10]. In addition, it is possible to build MSNs that react to internal or external stimuli, including pH, redox potential, temperature, magnetic field, or light; these are stimuli-responsive, or smart, drug delivery systems [11,12]. Multifunctional nanocarriers with such abilities have been shown to be more effective therapeutically than other formulations, lowering systemic toxicity, and with greater concentration at target sites [13,14].

The hybrid mesoporous silica nanoparticles (HMSNs) have recently been developed and this has added to the biomedical applications of MSNs [15]. These hybrid platforms combine the mechanical strength of silica and the pliability, degradability, and responsiveness of organic materials through the inclusion of the inorganic silica matrix with organic polymers, lipids, or metallic components [16,17]. For example, polymer-coated MSNs enable either pH-responsive or enzyme-induced drug release, whereas lipid-modified MSNs provide enhanced biocompatibility and prolonged circulation times [18,19]. The development of these hybrid systems is a milestone in the manufacture of the next-generation nanocarriers with enhanced therapeutic precision and clinical uses [14,20].

Despite their advantages, aspects such as mass production, repeatability, biodegradation, and long-term safety remain significant challenges in respect of their clinical application [21,22]. To ensure their safe use in human therapies, a thorough evaluation of their toxicity, biodistribution, and destiny in vivo is essential [23]. This review therefore aims to provide a detailed account of the synthesis procedures, structural characteristics, surface functionalization strategy, and biomedical uses of mesoporous silica and hybrid nanoparticles. Moreover, it discusses the new trends in stimuli-responsive nanocarriers, how they can be used to deliver drugs in a targeted fashion, and the safety issues that should be considered when conducting them in nanomedicine and pharmaceutics.

## 2. Synthesis of Mesoporous Silica and Hybrid Nanoparticles

Due to their architecture-tunable pore structure, vast surface area, and easy surface tunability, mesoporous silica nanoparticles (MSNs) have been an exciting new generation of nanocarriers in drug delivery [24]. Their physicochemical characteristics such as particle dimensions, shape, pore order, and surface reactivity directly depend on the synthesis pathway and these factors are vital in the loading efficiency, liberation rate, and biocompatibility [2,25,26]. A variety of synthetic methods have been developed such as the conventional sol–gel and Stöber methods to more advanced template mediated and hybridization methods [26]. The discussion that follows is a detailed explanation of the different methods with emphasis on their mechanism, strength, weaknesses, and emerging trends.

### 2.1. Chemical and Sol–Gel-Based Synthesis

#### 2.1.1. The Sol–Gel Process

The sol–gel method remains to be the building block of MSN. The silica precursor, such as tetraethyl orthosilicate (TEOS) or tetramethyl orthosilicate (TMOS), normally undergoes hydrolysis and a condensing reaction in an acid or base catalyzed alcohol–water mixture [27,28]. Introduced in 1968, the Stöber modification provides exhaustive control of monodispersibility of particles with parameters such as ammonia concentration, solvent polarity, and reaction temperature [29]. In the absence of defects, silanol groupings (Si-OH) are able to lead to the formation of siloxane-based (Si-O-Si) bridging groups and the formation of dense yet tunable silica networks [30]. The produced particles are highly stable, reproducible, and controllable in size with a range of 50 nm to several hundred nanometers [31,32]. However, the traditional sol–gel process typically produces non-porous or irregularly organized silica; hence, adding structure-directing agents was necessary to obtain ordered mesoporosity.

#### 2.1.2. Surfactant- or Template-Assisted Synthesis

Template-directed routes employ organic surfactants or macromolecules to structure the silica network into ordered mesopores [33]. Soft-templating involves the use of cationic surfactants like cetyltrimethylammonium bromide (CTAB) or triblock copolymers like Pluronic P123, which self-assemble into micellar arrays and act as scaffolds for silica precipitation [34,35]. When these micelles are hydrolyzed with TEOS, condensation continues, and the removal of the subsequent surfactant (either by calcination or solvent extraction) yields regular mesoporous channels with pore diameters usually in the range of 2–10 nm [24,36]. Hard-templating or nanocasting, on the other hand, uses pre-existing solid templates (e.g., polymer spheres, carbon particles, calcium carbonate, or silica colloids) [37]. Silica precursors penetrate the template pores, and the removal of templates creates hollow or core shell morphology beneficial for drug encapsulation and co-delivery applications [17,38]. Because leftover extraction might clog pores and compromise biocompatibility, template removal is still a crucial step. Advanced mild extraction methods such as supercritical CO_2_ extraction [39], microwave-assisted removal [40], and bio-enzymatic degradation of surfactants [41,42,43] have been developed to minimize structural collapse and residual contamination.

#### 2.1.3. Doping and Co-Condensation Approaches

**Figure 1:**
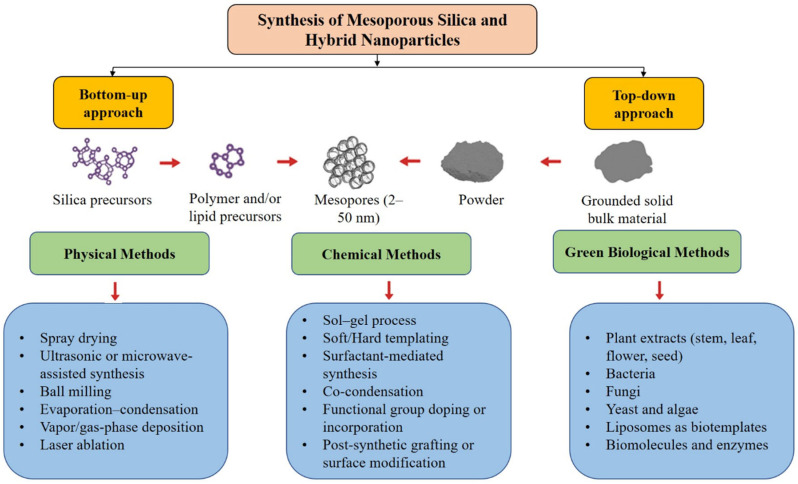
Schematic representation of mesoporous silica nanoparticle (MSN) synthesis approaches, including bottom-up (self-assembly and sol–gel) and top-down (fragmentation) methods used to produce silica and hybrid nanostructures.

To introduce chemical functionality during synthesis, the precursors of co-condensation are organosilane precursors (e.g., 3-aminopropyltriethoxysilane (APTES), mercaptopropyltrimethoxysilane (MPTMS)) along with TEOS in a single-step sol–gel reaction [44,45]. This guarantees a one-step distribution of functional groups on the silica matrix, which makes the silica more reactive to chemical conjugation or responses in the future [46]. Also, the metal ion doping (Fe^3+^, Mn^2+^, Zn^2+^) or incorporating fluorescent dyes into the silica structure provides magnetic or optical capability to image and treat diseases [47]. Overdoping can, nevertheless, break the geometry of the pores and reduce structural order [48]. Figure 1 gives an overview of the main MSN synthesis routes, both bottom-up and top-down, highlighting the impact of the synthesis method on particle morphology and physicochemical characteristics. Table 1 gives a comparative overview of the main synthesis methods for mesoporous silica and hybrid nanoparticles.

### 2.2. Post-Synthetic Surface Modification

#### 2.2.1. Surface Grafting and Functionalization

After the removal of the template, surface silanol groups facilitate the post-synthetic grafting of organosilanes with reactive moieties like amino, thiol, or carboxyl groups [60]. This allows further conjugation of targeting ligands, polymers, or drugs through covalent bonding [61]. As an illustration, APTES-modified MSNs enhance the conjugation of doxorubicin using electrostatic or amide bonds, and thiol-modified MSNs enhance redox-below disulfide bond conjugation [24,62]. Grafting therefore leaves space to adjust the surface charge and hydrophobicity to increase the drug carrier interaction.

#### 2.2.2. Polymer and Lipid Coating

MSNs can be further polymer-coated with polyethylene glycol (PEG), chitosan, poly(L-lactic acid), or pH-sensitive copolymer to increase biocompatibility and prevent a systemic immune response [63,64]. Polymer coating prevents the agglomeration of nanoparticles, reduces protein adsorption, and can be used to stimulate stimuli-responsive gating [65]. Similarly lipid–silica hybrids (LSHs), i.e., mesoporous cores surrounded by a lipid bilayer, mimic the structure of liposomes yet retain silica-stability and attain better hydrophobic drug pickup and increased bio-interface compatibility [66]. These inorganic-organic hybridized coats are effective in the combination of inorganic rigidity and the organic pliability [67].

#### 2.2.3. Gatekeeper and Stimuli-Responsive Systems

More complex functionalization involves the use of so-called gatekeeper molecules which can open the pore under physiological stimulation [64]. The pore entrances have polymer, cyclodextrin, or inorganic nanovalve caps which are liberated on particular initiators such as pH decrease, redox potentials, enzyme activity, temperature change, or light irradiation [68,69]. For instance, disulfide-bonded PEG caps are released in glutathione-rich tumor microenvironments, releasing the cargo selectively [70]. These intelligent systems play a central role in the attainment of spatiotemporal drug control [71].

### 2.3. Hybridization Strategies

#### 2.3.1. Polymer–Silica Hybrids

Hybrid nanoparticles of polymeric matrices interpenetrated by mesoporous silica retain the mechanical stability and high loading capacity of silica and the biodegradability and responsiveness of polymers [72]. Examples include poly(N-isopropylacrylamide) (PNIPAM)-MSNs with thermo-responsive swelling for on-demand release of drugs and chitosan-MSNs with mucoadhesive character for oral delivery [73,74].

#### 2.3.2. Lipid–Silica Hybrids

Lipid–silica hybrids combine a lipid bilayer or monolayer on a mesoporous core to produce biomimetic interfaces that allow cellular internalization and shield cargo within [75]. The lipid shell also enhances stealth properties by being resistant to opsonization and clearance. These LSHs have been applied in delivering anticancer drugs, siRNA, and hydrophobic antibiotics [75,76].

#### 2.3.3. Metal– or Magnetic Silica Hybrids

The insertion of metallic or magnetic cores (e.g., Fe_3_O_4_, Au, CuO) into mesoporous silica provides multifunctional hybrids that can perform dual imaging and therapy (theragnostic) [77,78]. Magnetic MSNs facilitate magnetically guided drug delivery and hyperthermia, and gold–silica hybrids allow photothermal or plasmonic effects for chemo-phototherapy [79,80]. Yet, maintaining interfacial stability and avoiding possible metal ion toxicity are ongoing research challenges. Recently, biomimetic hybrid MSNs coated or cloaked with natural membranes (e.g., erythrocyte, cancer cell, or exosome membranes) have emerged as a promising subclass providing immune-evasive and site-homing capabilities [81,82]. These designs combine the structural tunability of silica with biological identity and functionality [83].

### 2.4. Comparative Evaluation of Synthesis Approaches

Every pathway of synthesis has unique benefits and drawbacks. Sol–gel is inexpensive, low in cost, and readily scalable but has limited control over pore ordering. Template-based methods have better pore architecture and uniformity but at the expense of extra steps to remove surfactant [84]. Co-condensation and hybridization combine multifunctionality but potentially at the expense of reproducibility and economy of production [85,86]. The choice of method should thus be based on the required application, high drug loading, stimuli responsiveness, or clinical scalability.

### 2.5. Parameters Affecting MSN Formation

**Figure 2:**
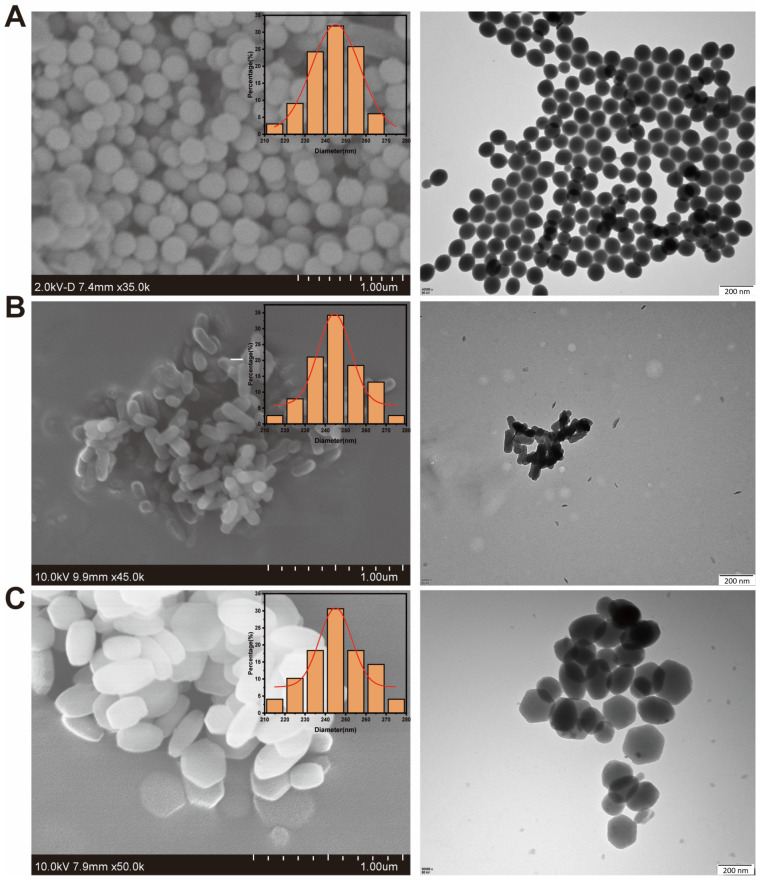
Morphology of mesoporous silica nanoparticles (MSNs) with different shapes. Scale bar = 200 nm. Shown are SEM images with corresponding size distributions and TEM images of (**A**) MSN-S, (**B**) MSN-R, and (**C**) MSN-H. The figure is reproduced exactly as published in the original article [109]. Reproduced from [109]. Pharmaceutics, **16**, 632. © 2024 by the authors. Published by MDPI, Basel, Switzerland, and distributed under the terms of the **Creative Commons Attribution (CC BY 4.0) License**.

The morphology and pore characteristics of MSNs are governed by multiple factors (Figure 2):

- pH and Catalyst Concentration: The basic conditions promote hydrolysis and condensation and give smaller monodisperse particles; acidic media prefer large, irregular morphologies [87].
- Surfactant Concentration: This defines the size of the micelle and therefore the size of the pore; too much surfactant may result in aggregation [88].
- Temperature and Reaction Time: They increase the kinetics of condensation with high temperatures but can kill the mesostructure when not controlled [89].
- Solvent Ratio: Modulates precursor solubility and micellar organization [90].
- Calcination Conditions: Affect framework integrity; overly high temperatures shrink pore volume or induce sintering [91].

Adjusting these parameters guarantees consistent drug delivery performance and the repeatable synthesis of MSNs and their hybrids. While diverse synthesis routes provide flexibility in tailoring pore structures, direct comparisons of scalability and reproducibility remain limited. Large-scale, defect-free production of MSNs under GMP-compatible conditions is still an unmet challenge that hinders clinical translation.

## 3. Physicochemical Characterization of Mesoporous Silica and Hybrid Nanoparticles

Extensive physicochemical analysis is critical in establishing the structure–function relationship of mesoporous silica nanoparticles (MSNs) and their hybrid counterparts [24]. Functional attributes such as particle size, surface morphology, pore structure, surface chemistry, and colloidal stability significantly influence drug loading, release profile, biodistribution, and biological interactions [7,92,93].

### 3.1. Particle Size, Pore Size, and Surface Area Analysis

In order to determine biodistribution, cellular uptake, and clearance, MSN particle size and size distribution are crucial. Normally, dynamic light scattering (DLS) and transmission electron microscopy (TEM) are employed together to achieve precise particle dimensions [94,95]. DLS quantifies the hydrodynamic diameter in colloidal suspension, which includes aggregation and surface coating effects, while TEM gives high-resolution particle core images [96]. For drug delivery, particle sizes within the 50–200 nm range are mostly used to attain improved permeation and retention (EPR) effects and to prevent fast renal clearance [97,98].

Pore size and surface area are typically characterized by nitrogen adsorption desorption isotherms measured using the Brunauer–Emmett–Teller (BET) and Barrett–Joyner–Halenda (BJH) models [99]. Total surface area is measured using the BET method from multilayer gas adsorption, and pore diameter distribution and volume are calculated from the desorption branch using the BJH model. MSNs generally display Type IV isotherms with H1- or H2-type hysteresis loops typical of mesoporous materials (2–50 nm pores) [36,100]. Reported BET surface areas are generally between 500 and 1200 m^2^ g^−1^ and vary with synthesis parameters, the type of surfactant, and post-treatment [57,101]. These directly influence the drug adsorption capacity and diffusion through the pore channels.

### 3.2. Morphological and Structural Characterization

#### 3.2.1. Transmission and Scanning Electron Microscopy (TEM and SEM)

TEM offers nanoscale imaging of particle shape, size, and internal pore order, allowing hexagonal or radial mesoporous channel observation typical of MCM-41-like morphology [2,102]. SEM, by contrast, provides surface topology, aggregation state, and general morphology information. Coupling the two enables the precise measurement of monodispersity and morphology (spherical, rod-like, or worm-like) [85,103]. In hybrid nanoparticles, TEM may detect the core–shell structure, polymer coating thickness, or embedded metallic domains [104,105]. Representative transmission and scanning electron micrographs of mesoporous silica nanoparticles (Figure 2) show uniform spherical and rod-like morphologies with well-ordered mesopore channels, confirming the MCM-41-type structure typical of drug delivery-grade MSNs. Similar morphologies have been consistently reported in previous studies [26,106,107,108].

#### 3.2.2. Atomic Force Microscopy (AFM) and High-Resolution Imaging

AFM provides three-dimensional surface images in air or liquid conditions, giving information on the surface roughness and coating homogeneity, particularly for polymer- or lipid-functionalized MSNs [110]. HRTEM and cryo-TEM further explain mesostructured ordering and hybrid interface arrangement [111,112].

### 3.3. Porosity and Pore Structure Evaluation

Nitrogen adsorption–desorption is the established technique for measuring pore volume, surface area, and pore size distribution [113]. The typical Type IV isotherm validates mesoporosity, whereas hysteresis loop topology reveals pore shape (H1 = cylindrical, H2 = ink-bottle, H3 = slit-like) [114,115]. Supplementary small-angle X-ray scattering (SAXS) gives information about the long-range order as well as the lattice periodicity of mesoporous architectures [116,117]. In hybrid systems, changes in pore size or decreases in surface area following polymer/lipid coating validate the successful surface modification and partial pore blockade [118]. Mercury intrusion porosimetry is sometimes applied to macroporous hybrid composites, but might destroy fragile structures [119,120].

### 3.4. Surface Chemistry and Functional Group Analysis

#### 3.4.1. Fourier Transform Infrared Spectroscopy (FTIR)

FTIR spectroscopy detects characteristic vibrational bands for Si–O–Si asymmetric stretching (~1080 cm^−1^), symmetric stretching (~800 cm^−1^), and bending (~460 cm^−1^), verifying siloxane network formation [121,122]. Broad O–H stretching at 3400 cm^−1^ indicates surface a silanol group presence. New peaks (e.g., –NH_2_ at 1560–1650 cm^−1^, –SH near 2550 cm^−1^, or C=O at 1720 cm^−1^) after functionalization verify the successful grafting of organic moieties [123,124]. FTIR is therefore a critical instrument to confirm chemical alterations and interactions among silica and hybrid materials.

#### 3.4.2. X-Ray Photoelectron Spectroscopy (XPS)

XPS provides elemental composition and chemical-state information on the MSN surface, typically to 5–10 nm in depth [125,126]. Silicon 2p (~103 eV) and oxygen 1 s (~532 eV) peaks denote the silica backbone, with other signals (C 1 s, N 1 s, S 2p) indicating grafted functional groups or polymer/lipid sheathing [127,128]. Quantitative XPS enables the calculation of functional group density and hybrid composition ratio, enabling precise control over surface chemistry [129,130].

### 3.5. Colloidal Stability and Surface Charge

The zeta potential (ζ-potential), as measured by electrophoretic light scattering, defines the charge at the surface and the electrostatic stability of MSN suspensions [131,132]. Silica nanoparticles typically possess negative ζ-potential values (−30 to −50 mV) due to deprotonated silanol groups. Amine or polymer group functionalization changes ζ-potential to positive or neutral values, affecting cellular uptake and serum stability [133,134]. Nanoparticles with |ζ| > 30 mV tend to have good colloidal stability as a result of electrostatic repulsion [135]. DLS measurements supplement zeta potential analysis by showing changes in the hydrodynamic diameter under physiological conditions. In hybrid systems, polymer or lipid shells tend to lower the magnitude of ζ-potential and hence nonspecific adsorption of protein and enhance hemocompatibility [136,137].

### 3.6. Crystallinity and Structural Order

X-ray diffraction (XRD) and small-angle X-ray scattering (SAXS) are commonly used to evaluate the structural order of mesoporous silica [117,138]. Low-angle XRD patterns show characteristic reflections at 2θ = 2–4° corresponding to (100), (110), and (200) planes, establishing hexagonal (p6 mm) symmetry characteristic of MCM-41-type material [104,139]. Broad peaks at increased angles demonstrate the amorphous character of silica. The attenuation or displacement of low-angle peaks in hybrid materials can result from the partial filling of pores by polymers or lipids [140,141]. Supplemental thermogravimetric analysis (TGA) measures organic loading and the stability of the coating, whereas differential scanning calorimetry (DSC) assesses the thermal transitions of hybrid components [142,143]. When taken as a whole, these techniques demonstrate mesostructure maintenance after alteration and provide information on thermal stability.

### 3.7. Comprehensive Assessment and Relevance to Drug Delivery

The systematic combination of the methods described above yields efficient characterization of MSN-based systems. TEM and nitrogen sorption, for instance, confirm physical mesoporosity; FTIR and XPS confirm surface chemistry; and DLS/zeta potential analysis confirms colloidal behavior in biofluids [144,145]. Insights into these parameters are essential to ensure that physicochemical properties are predictive of drug loading capacity, release profiles, biocompatibility, and in vivo efficacy. Current reports bring to the fore the need to correlate release rate constants with BET surface area and pore diameter, paving the way for rational nanocarrier design [146,147]. Although extensive characterization data exist, there remains a lack of standardized protocols to correlate physicochemical metrics such as BET surface area and zeta potential with in vivo pharmacokinetics and toxicity outcomes. Future studies should aim to establish cross-platform comparability and reproducibility.

## 4. Functionalization and Stimuli-Responsive Designs

The functionalization of mesoporous silica nanoparticles (MSNs) is a key process in the design of their physicochemical, biological, and therapeutic behavior [24]. The presence of terminal surface silanol (Si–OH) groups allows facile covalent modification by organic ligands, polymers, and biomolecules, transforming MSNs into multifunctional, targeted, and responsive nanoplatforms [92,148]. The functionalization improves colloidal stability, supports active targeting, and allows controlled drug release under particular physiological conditions [149]. The functionalization approaches can be generally categorized under surface ligand grafting, polymer/lipid coatings, and stimulus-responsive gating mechanisms, frequently being incorporated into theragnostic or co-delivery platforms for multifunctional applications [150].

### 4.1. Surface Grafting of Targeting Ligands

#### 4.1.1. Ligand Functionalization for Active Targeting

Active targeting capitalizes on the special interactions between MSN surface-bound ligands and the overexpressed receptors on target cells. Functionalized ligands such as antibodies, peptides, aptamers, folic acid, or carbohydrates are covalently attached to amine- or carboxyl-functionalized MSNs through carbodiimide (EDC/NHS) or click-chemistry reactions [5,151,152]. For instance, folic acid (FA) is commonly used to target folate receptor-positive cancer cells, promoting uptake and cytotoxic potency over non-targeted carriers [92,153]. Likewise, RGD peptides target integrin receptors that are overexpressed on tumor vasculature and induce receptor-mediated endocytosis [154].

Aptamer-conjugated MSNs have attracted interest owing to their strong binding affinity and low immunogenicity. Aptamers can be grafted using thiol or amine linkers for DNA or RNA and show selective binding towards cancer biomarkers like MUC1 or HER2 [155,156]. Antibody-functionalized MSNs, though slightly larger in size, show great precision in targeting, such as for trastuzumab-MSNs to target HER2-positive breast cancer [157,158]. These biofunctional MSNs show much higher therapeutic indices with reduced systemic toxicity [159]. Despite their selectivity, aptamers may suffer from limited nuclease stability and require chemical modification (e.g., 2′-fluoro or 2′-O-methyl substitutions) to remain intact in biological fluids [160]. Likewise, antibody conjugation involves complex bioconjugation chemistry, potential loss of binding affinity, and batch-to-batch variability [161].

#### 4.1.2. Multivalent and Dual-Ligand Systems

Current advances focus on multivalent functionalization, in which several ligands are linked to enhance binding avidity and cellular uptake [2]. Dual-ligand architectures (e.g., FA and RGD or antibody and aptamer) permit the concurrent recognition of two receptor subtypes, mitigating tumor heterogeneity and receptor downregulation [162,163]. Optimizing ligand density and spatial arrangement is still necessary to ensure colloidal stability and prevent steric obstruction [104].

### 4.2. Polymer Functionalization and Coatings

#### 4.2.1. Polyethylene Glycol (PEG) and Stealth Properties

Polymer coatings confer stability and “stealth” characteristics to MSNs, inhibiting aggregation and opsonization. PEGylation, the covalent binding of polyethylene glycol (PEG), creates a hydrophilic steric shield that decreases protein adsorption and enhances circulation half-life [164]. PEG-coated MSNs show decreased macrophage uptake and increased tumor site accumulation through the improved permeability and retention (EPR) effect [165]. Thiol–PEG or silane–PEG linkers also offer the possibility for subsequent conjugation with targeting ligands or stimuli-responsive moieties [64,166].

#### 4.2.2. Stimuli-Responsive Polymers

Smart polymers allow environmental responses to pH, redox potential, temperature, or enzyme changes. pH-sensitive polymers like poly (acrylic acid), chitosan, or poly (β-amino ester) ionize or dissolve in acidic tumor or endosomal environments, initiating cargo release [22]. Redox-responsive polymers with disulfide linkages break in glutathione (GSH)-rich cytosolic environments, enabling intracellular drug release [167]. Poly(N-isopropylacrylamide) (PNIPAM) thermosensitive coatings contract above their lower critical solution temperature (LCST), releasing encapsulated molecules upon mild hyperthermia [168]. Stimuli-sensitive polymers can also act as gatekeepers that block or open MSN pores upon physiological signals [169].

### 4.3. Stimuli-Responsive Mechanisms

MSNs can be designed to respond to various internal (pH, redox, enzyme) and external (light, magnetic field, temperature) stimuli. These “smart nanocarriers” allow for the controlled and site-specific release of therapeutic loads, reducing off-target effects [21,170].

#### 4.3.1. pH-Responsive Systems

pH difference between healthy tissues (pH 7.4) and tumor microenvironments (pH 6.5–5.0) is a natural impetus for self-controlled drug release [171]. Acid-cleavable linkers like hydrazone, imine, or acetal bonds are incorporated to connect drug molecules or polymer caps that break under acidic pH [172]. For instance, hydrazone-conjugated doxorubicin-MSNs deliver the drug selectively in lysosomes, enhancing therapeutic specificity [21,173].

#### 4.3.2. Redox-Responsive Systems

Tumor and intracellular compartments contain higher concentrations of GSH (2–10 mM) compared to extracellular fluids (2–20 µM). Disulfide-bonded polymer- or cyclodextrin-capped MSNs release their loaded substance after disulfide bond cleavage by GSH [13,174]. This strategy guarantees intracellular drug release only after cell uptake [175,176].

#### 4.3.3. Enzyme-Responsive Systems

Pathologically overexpressed enzymes, like matrix metalloproteinases (MMPs), esterase, or phospholipase, can initiate the degradation of polymer coatings or gatekeeper linkers [177]. MMP-cleavable peptide linkers have been effectively loaded onto MSNs to attain selective release within tumor microenvironments [178,179].

#### 4.3.4. Light- and Temperature-Responsive Systems

**Figure 3:**
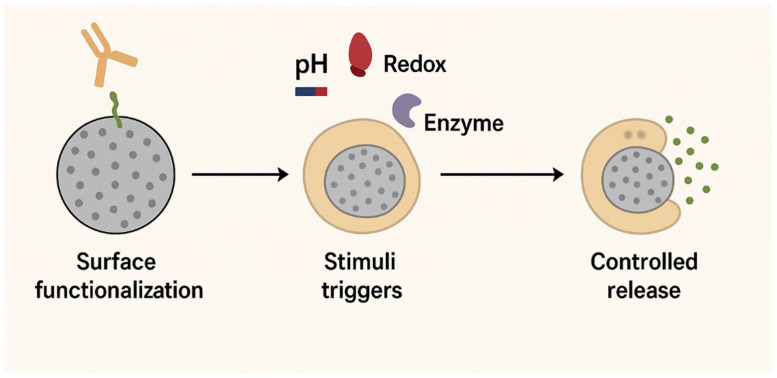
Schematic representation of surface functionalization and stimuli-responsiveness mechanisms in mesoporous silica nanoparticles (MSNs). The schematics represent surface grafting with polymers or antibodies for stability and targeting followed by stimuli-activated responses such as pH, redox, and enzyme sensitivity leading to site-specific and controlled release of drugs.

Photoresponsive systems utilize photocleavable linkers (e.g., o-nitrobenzyl derivatives) or plasmonic nanoparticles (e.g., Au–MSNs) that heat locally when irradiated with near-infrared (NIR), causing pore opening and on-demand release [104,180]. Temperature-sensitive MSNs based on PNIPAM or lipid–silica hybrids exhibit reversible expansion/contraction behaviors, allowing thermally induced cargo expulsion [181,182]. These systems are especially useful for chemo-photothermal combination therapies. As illustrated in Figure 3, the functionalization of MSNs with polymers, ligands, and responsive caps enables stimuli-triggered and site-specific drug delivery.

Table 2 details illustrative examples of surface functionalization and response to stimuli mechanisms for improved targeted or controlled drug delivery with MSNs.

### 4.4. Gatekeeper Strategies and Molecular Valves

Gatekeeper structures physically occlude MSN pore entrances until opened by an initiating stimulus. Different caps such as nanoparticles, supramolecular complexes, or polymers are attached by cleavage-sensitive bonds [204]. Supramolecular cyclodextrin adamantane host–guest interaction-based caps, mesoporous magnetic or quantum dot-based core shell valves, and acid-cleavable bond-based polymer gates are representative strategies [23]. These “molecular valves” accurately block early drug leakage and deliver precise spatiotemporal control [205,206].

For example, in 2019, β-cyclodextrin-capped MSNs were developed that remained sealed at physiological pH but opened in acidic tumor microenvironments, effectively releasing doxorubicin [207,208]. Likewise, redox-responsive PEG gates connected via disulfide bridges allowed for controlled drug delivery in intracellular reductive environments [209,210].

### 4.5. Co-Delivery and Theragnostic Hybrid Systems

The integration of therapeutic and diagnostic (theragnostic) activities within a single MSN platform is the next big thing in nanomedicine. Co-delivery systems facilitate the co-transport of multiple agents of chemotherapeutics, genes, or imaging probes via a single nanocarrier [24,211].

#### 4.5.1. Drug/Gene Co-Delivery

MSNs possess the ability to carry small-molecule drugs and nucleic acids (DNA, siRNA, or miRNA) concurrently via surface electrostatic interactions and pore loading [97,212]. For instance, chitosan-coated MSNs co-delivering doxorubicin and siRNA exhibited synergistic growth inhibition of tumors and gene silencing [213]. These systems bypass multidrug resistance and enable combination chemo-gene therapy [214].

#### 4.5.2. Theragnostic Hybrids

In theragnostic MSNs, diagnostic moieties like fluorescent dyes, magnetic nanoparticles (Fe_3_O_4_), or gold nanoclusters are embedded to facilitate the real-time imaging of drug release and biodistribution [215,216]. For example, folic acid-functionalized Fe_3_O_4_@MSNs display dual MRI and fluorescence imaging ability as well as targeted drug delivery [217]. Gold–silica hybrids facilitate simultaneous photothermal ablation and chemotherapy under NIR irradiation [218,219]. These systems represent the intersection of therapy and diagnosis, facilitating image-guided precision medicine.

### 4.6. Challenges and Future Outlook

Although unprecedented advances have been achieved, there remain some challenges in achieving reproducibility and homogeneous functionalization. Technical hurdles of the control of ligand density, coating of the polymer uniformly, and colloidal stability upon complexation are crucial [220,221]. Moreover, the bulk fabrication of stimuli-responsive MSNs for translational applications necessitates extensive biocompatibility testing and stability analysis under physiological conditions [222]. Subsequent advancements will integrate multi-responsive platforms that react to internal and external stimuli which are integrated and combined to reach closer towards intelligent nanoplatforms for targeted therapy [223]. Despite impressive progress in stimuli-responsive systems, most studies remain proof-of-concept with limited in vivo validation. The scalable synthesis of multifunctional coatings and regulatory reproducibility for complex hybrid systems are still unresolved obstacles to clinical use.

## 5. Drug Loading and Release Mechanisms

The potential of mesoporous silica nanoparticles (MSNs) to load, store, and deliver therapeutic cargo efficiently under programmed conditions is the key to their application in drug delivery. Their large surface area, homogeneous mesoporous structure, and adjustable pore size (2–50 nm) provide adaptable loading mechanisms through physical adsorption, covalent conjugation, or encapsulation within hybrid matrices [92,148]. The loading mechanism needs to be optimized to attain prolonged, site-specific, and biocompatible drug release with reduced premature leakage and systemic side effects. Physicochemical interactions, pore structure, surface modification, and external/internal stimuli regulate the drug release kinetics from MSNs [49].

### 5.1. Drug Loading Mechanisms

#### 5.1.1. Adsorption Within Mesopores

The simplest and most extensively used loading approach is physical adsorption. Drugs permeate into the mesopores and stick to the silica surface by hydrogen bonding, van der Waals forces, and electrostatic forces [2]. The adsorption process is usually carried out by submerging the nanoparticles in a drug stock solution under mild agitation or under vacuum impregnation [53]. The surface area, pore size, and chemical affinities between drug molecules and silanol groups influence the adsorption efficiency. Hydrophilic medications interact through hydrogen bonding, whereas hydrophobic medications are more retained through van der Waals and hydrophobic interactions [224].

The simplicity and reproducibility of adsorption make it the best choice for small-molecule drugs such as doxorubicin, paclitaxel, or ibuprofen [225]. But if not controlled, desorption leads to the burst release, thereby causing immediate leakage of the drug before it reaches the target site [226]. Surface functionalization with polymers (PEG, chitosan) and the pore-capping systems described in Section 2.2 and Section 4.2 are the primary strategies to minimize burst release, yielding more sustained and predictable diffusion kinetics [165]. For this reason, surface modification or pore-capping techniques are commonly applied to combat this challenge and provide controlled diffusion.

#### 5.1.2. Covalent Conjugation

Covalent attachment consists of chemically bonding the drug molecules to functional groups on the silica surface with biodegradable linkers like ester, amide, or disulfide bonds [227]. The approach stabilizes loading and offers reproducible release initiated by bond cleavage in definite physiological conditions. For example, doxorubicin has been covalently attached to amino-functionalized MSNs using hydrazone linkers that hydrolyze under acidic pH to achieve site-specific release in tumor microenvironments [223]. Likewise, redox-sensitive disulfide bridges enable the GSH-mediated intracellular drug release [26].

Covalent conjugation provides higher release kinetic control and prevents leakage before time, although it tends to need multistep synthesis and exact linker optimization [9].

#### 5.1.3. Encapsulation Within Hybrid Matrices

In hybrid nanocarriers, drugs are delivered encapsulated in polymer–, lipid–, or metal–silica matrices. The polymer–silica hybrid platforms (e.g., chitosan-coated or PEG–MSNs) improve biocompatibility and regulate drug diffusion via the swelling or degradation of the polymer. Lipid–silica hybrids create core shell morphologies that enhance hydrophobic drug loading and inhibit burst release owing to the hydrophobic lipid barrier. Encapsulation also allows co-loading of multiple agents, which safeguards sensitive biomolecules like siRNA or proteins from degradation [64].

### 5.2. Release Behavior and Kinetic Profiles

Release of the drug from MSNs typically takes place by diffusion through mesopores, surface site desorption, or breakage of functional linkages. The release pattern can be a burst, sustained, or triggered depending on the strength of the interaction and surface chemistry [228].

#### 5.2.1. Burst vs. Sustained Release

Burst release is due to loosely adsorbed drug molecules at the outer surface or at the pores’ mouth, leading to a temporary high-concentration release period. Although useful for instantaneous therapeutic response (e.g., antimicrobial agents), it is toxic for cytotoxic drugs [223].

In contrast, extended release occurs when drugs are strongly encapsulated in mesopores or covalently bonded to the surface to release slowly over hours or days. Surface grafting with polymers such as PEG, PAA, or chitosan easily reduces burst effects and allows sustained, zero-order release kinetics. Lipid hybridization or grafting with stimuli-responsive polymers provides additional control, extending release time while retaining bioactivity [229].

#### 5.2.2. Controlled and Triggered Release

Controlled release refers to systems that release drugs in response to internal or external stimulation with the benefit of spatial and temporal precision. Internal stimuli are pH gradients, redox potential, and enzyme activity, whereas external stimuli are magnetic fields, light, or heat [19].

**Figure 4:**
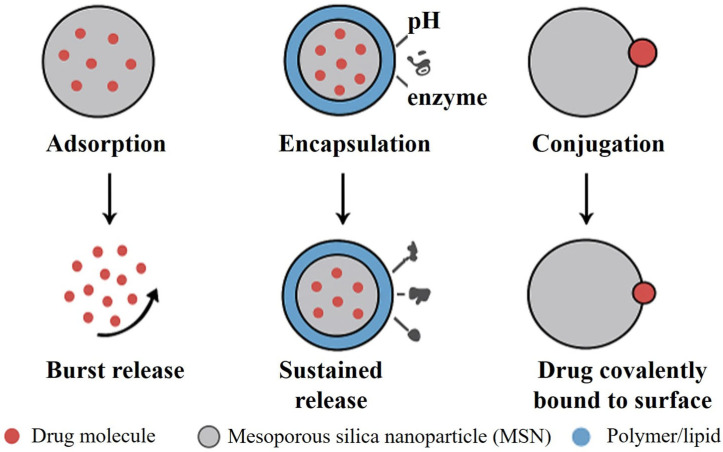
Schematic illustration of drug loading and release in mesoporous silica and hybrid nanoparticles, showing adsorption within mesopores, encapsulation in polymer/lipid shells, and covalent surface conjugation, with burst and sustained release profiles controlled by stimuli such as pH, redox, or enzymes.

For example, pH-sensitive hydrazone spacers are hydrolyzed in acidic tumor environments, and GSH-sensitive disulfide bonds are cleaved in reductive cytosolic conditions, both enabling controlled intracellular release. Photoresponsive MSNs that contain photosensitive functionalities (e.g., azobenzene or coumarin) enable on-demand release upon NIR illumination. Such systems are especially useful in cancer treatment, minimizing systemic toxicity [223]. Figure 4 schematically illustrates the drug loading and release mechanisms, emphasizing adsorption, encapsulation, and conjugation pathways along with burst and sustained release profiles.

### 5.3. Mathematical Models of Drug Release

Quantitative modeling of release kinetics is important to predict diffusion and degradation processes. A number of empirical and semi-empirical models have been used for MSNs [230].

- Zero-Order Model:

Assumes a fixed drug release rate regardless of concentration and is well-suited to keep plasma levels constant [231].

Qt = Q0 + k0t

where Qt is the released drug at time t, and k0 is the zero-order constant.

- Higuchi Model:

Accounts for diffusion-controlled release from a porous matrix, where drug flux reduces with time [232].

Qt = kHt1/2

where kH is the Higuchi dissolution constant.

- Korsmeyer–Peppas Model:

Used for complex systems exhibiting non-Fickian behavior, with both diffusion and polymer relaxation contributions [233].

Qt/Q∞ = ktn

where n identifies the mechanism: n < 0.45 (Fickian), 0.45 < n < 0.89 (anomalous), n ≥ 0.89 (Case-II transport).

These models enable physicochemical parameters to be correlated with experimental data, facilitating the optimization of MSN-based delivery systems for reproducible therapeutic performance. Unlike conventional tablet- or polymer-based formulations, drug release kinetics from mesoporous silica nanoparticles (MSNs) are governed by nanoscale-specific parameters. The release rate is affected by the pore diameter, volume, and surface area, which determine the diffusion pathway, as well as by surface functionalization, drug–matrix interactions, and external coatings that can impose diffusion barriers. Consequently, while empirical models such as Higuchi or Korsmeyer–Peppas are useful for describing overall release trends, they must be interpreted considering these nanostructural characteristics. The drug release from MSNs often exhibits a combination of diffusion through mesopores, desorption from surface sites, and, in some systems, stimuli-responsive behaviors. These features distinguish MSN-based release mechanisms from those of conventional dosage forms and provide a more mechanistic understanding of their release kinetics [4,26,151,234].

### 5.4. Co-Delivery and Multifunctional Systems

The integration of multiple therapeutic and diagnostic agents within a single MSN system enables synergistic effects and multimodal therapy [24].

#### 5.4.1. Drug and Gene Co-Delivery

MSNs have been effectively used for the simultaneous delivery of small-molecule therapeutics and nucleic acids, capitalizing on their high drug loading capacity and tunable surface charge. Cationic coatings (e.g., chitosan or polyethyleneimine) enable the electrostatic adsorption of negatively charged siRNA or DNA while preserving pore loading for hydrophobic drugs [24]. For example, dual-loaded MSNs with doxorubicin and Bcl-2 siRNA exhibited synergistic apoptosis in cancer cells, bypassing multidrug resistance [21].

#### 5.4.2. Drug and Imaging Agent Co-Delivery

Theragnostic MSNs integrate therapy and imaging functionality by incorporating fluorescent dyes, magnetic nanoparticles (Fe_3_O_4_), or quantum dots into the silica matrix. The hybrids facilitate real-time visualizations of biodistribution, intracellular drug release, and therapeutic effects. The simultaneous co-loading of chemotherapeutic drugs with imaging probes enables non-invasive monitoring and dosage adjustment in vivo [216].

### 5.5. Factors Influencing Drug Loading and Release

A number of physicochemical parameters play a role in determining drug loading and release efficiency:

- Pore Volume and Size: Large pores are used to load macromolecules (siRNA, proteins) while diffusion is limited in small pores [26].
- Surface Chemistry: Drug–carrier interactions are controlled by functional groups; amine-functionalized MSNs facilitate acidic drug loading [24].
- pH and Ionic Strength: Controls ionization status of silica and drug molecules, affecting electrostatic adsorption [235].
- Solvent polarity and viscosity: Control rates of diffusion and solubility during loading and release [224].

The optimization of these parameters allows for the rational design of MSN-based delivery systems with the anticipated pharmacokinetics and maximized therapeutic efficacy.

### 5.6. Summary and Outlook

MSNs offer a very responsive platform for controlled and stimulus-responsive drug delivery. Choosing suitable loading and release mechanisms is determined by the drug characteristics, desired pharmacokinetics, and target tissue environment. Future developments are likely to center on multi-agent co-delivery, personalized release kinetics, and in vivo predictive modeling, facilitating the clinical translation of MSN-based nanomedicines [236]. While numerous polymer and lipid coatings enhance circulation time and reduce burst release, comparative pharmacokinetic data across formulations remain scarce. Establishing quantitative relationships between surface chemistry, release kinetics, and therapeutic efficacy will be critical for rational MSN design.

## 6. Biomedical Applications and Case Studies

**Figure 5:**
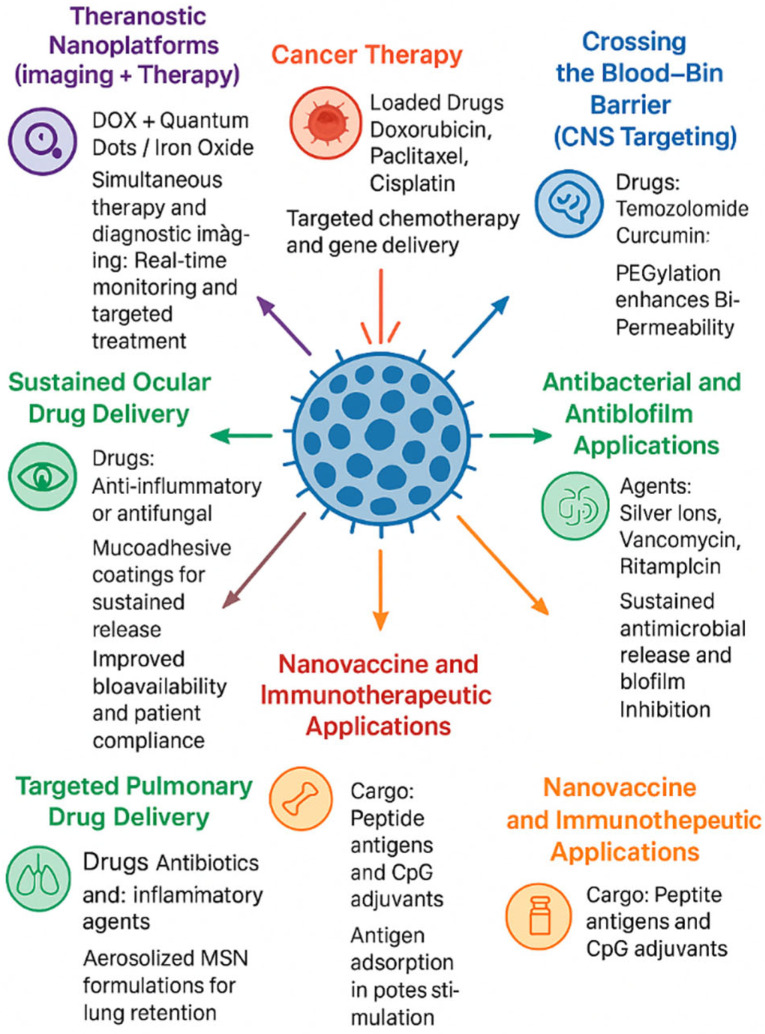
Biomedical applications of mesoporous silica and hybrid nanoparticles, highlighting their use in targeted cancer therapy, CNS drug delivery, antimicrobial and vaccine delivery, bone and ocular targeting, and multifunctional theragnostic systems.

Mesoporous silica nanoparticles (MSNs) and hybrid derivatives have been assessed for a wide range of biomedical applications due to their high loading capacity, tailorable release behavior, and easy surface chemistry. This section outlines the notable application areas of cancer therapy, central nervous system (CNS) delivery, antimicrobial approaches, vaccine/immunotherapy platforms, and other targeted delivery pathways and presents representative recent examples, performance metrics, and challenges to be met. As shown in Figure 5, MSNs have been successfully adapted across a diverse range of biomedical applications, including cancer therapy, antimicrobial systems, and nano-vaccine delivery. Table 3 compiles the recent applications of mesoporous silica and hybrid nanoparticles in diverse biomedical fields reported.

### 6.1. Cancer Therapy (Chemotherapy, Gene Delivery)

#### 6.1.1. Chemotherapeutic Delivery

MSNs find extensive use for the delivery of cytotoxic drugs (e.g., doxorubicin, paclitaxel) due to their high drug load ability and controlled release. Surface functionalization (e.g., folate, RGD peptides, antibodies) allows the active targeting to cancer cells, while responsive caps (pH, redox, enzyme) minimize off-target leakage and systemic toxicity. In vivo research indicates the enhanced tumor uptake and favorable therapeutic index over free drugs; for example, pH-sensitive MSN doxorubicin systems demonstrated greater tumor regression with less cardiotoxicity in murine xenograft models [223,255]. Performance metrics are drug encapsulation efficiency (>50–80% for most small molecules), extended release over days, and enhanced median survival in treated groups.

#### 6.1.2. Gene and Nucleic Acid Delivery

Gene and nucleic acid delivery with MSNs exploits the electrostatic complexation of negatively charged nucleic acids (DNA, siRNA, miRNA) onto cationic or polymer-modified surfaces such as PEI-MSNs or chitosan-MSNs [256]. The co-delivery of chemotherapeutics (e.g., DOX) and siRNA has shown synergistic effects in reversing multidrug resistance and improving transfection efficiency [196,257]. Critical parameters include particle charge, protection from nuclease degradation, and endosomal escape mechanisms.

### 6.2. CNS/Brain Delivery (Crossing the BBB)

Translocation through the blood–brain barrier (BBB) is a significant challenge; MSNs have been designed to overcome it through various approaches: (i) surface modification with BBB-penetrating ligands (transferrin, angiopep-2), (ii) use of receptor-mediated transcytosis, (iii) nasal delivery for nose-to-brain targeting, and (iv) temporary BBB modulation (e.g., targeted ultrasound) in combination with MSN delivery [258,259]. Preclinical findings show enhanced brain accumulation (2–6-fold) and therapeutic effects in glioma and neurodegenerative disease models when MSNs are surface-functionalized with BBB-targeting peptides. Nevertheless, issues regarding long-term retention, brain parenchymal clearance, and neuroinflammation need intense chronic toxicity studies prior to translation.

### 6.3. Antimicrobial/Antibacterial Delivery

MSNs provide sites for local and systemic antimicrobial treatment through facilitating high loci genic levels of antibiotics, controlled drug release, and combination therapy (antibiotic + metal ion or antimicrobial peptide). Surface-modified antibiotic-loaded hybrid MSNs (e.g., vancomycin, ciprofloxacin) with chitosan or cationic peptides demonstrate increased antibacterial efficacy and penetration into biofilms and decreased minimum inhibitory concentrations (MICs) against multidrug-resistant strains in in vitro and in vivo models of wounds [260,261]. MSNs are able to also deliver bacteriophages, antimicrobial peptides, or photosensitizers for photodynamic antimicrobial therapy. The major limitations are to achieve selective microbial toxicity with no host cell damage and to determine clearance pathways for repeated topical or systemic administration.

### 6.4. Vaccine and Immunotherapy Delivery

MSNs are used as antigen/adjuvant carriers to enhance antigen stability, presentation, and adjuvanticity. Their porosity allows for the co-loading of protein antigens and immunostimulatory molecules (CpG, MPLA), while the surface chemistry regulates antigen display and targeting to antigen-presenting cells (APCs). Recent preclinical vaccine research shows greater humoral and cellular immune responses than soluble antigen, with encouraging outcomes in influenza, cancer neoantigen, and bacterial antigen models [247,248]. In addition, MSNs have also been used to locally deliver immune checkpoint inhibitors or cytokines to the tumor environment, enhancing antitumor immunity. Favorable safety profiles are usually observed in short-term research, but immune-modulatory risks (e.g., undesirable systemic inflammation) need to be determined in longer-term models.

### 6.5. Other Targeted Delivery (Bone, Lung, Ocular)

- Bone targeting: Bisphosphonate or targeting peptide-decorated MSNs have been utilized for the osteotropic delivery of anticancer agents or growth factors and antibiotics for bone metastases and osteomyelitis. Local drug exposure is enhanced with controlled release and increased bone affinity while minimizing systemic dosing [262].
- Pulmonary delivery: Spray-dried or aerosolized inhalable MSN formulations for lung cancer or lung infections utilize high surface area and tunable aerodynamic diameter; mucopenetration is enhanced and macrophage clearance minimized with polymer coatings [263].
- Ocular delivery: MSNs offer controlled release in ocular spaces (conjunctiva, vitreous), counteracting the fast drainage and short residence time of traditional eye drops; mucoadhesive coatings (chitosan) enhance retention and therapeutic contact [264].

Every route requires customized considerations (particle size, surface hydrophobicity, sterility, and formulation stability) and regulatory safety testing appropriate to route-specific barriers.

### 6.6. Antimicrobial Spectrum: Anti-Biofilm, Antiviral, and Antiparasitic

Besides the antibacterial action, mesoporous silica nanoparticles (MSNs) possess an extensive antimicrobial activity with anti-biofilm, antiviral, and antiparasitic activity, which is greatly extended by the tunable surface chemistry, generation of reactive oxygen species (ROS), and the ability to deliver antimicrobial or immunomodulatory agents as carriers.

Anti-biofilm activity: Silver, zinc oxide, or the cationic polymer-engineered surface of MSNs can penetrate bacterial biofilms and destabilize extracellular polymeric substance (EPS) matrices [265]. The surface area enables ion release and the mechanical breakdown of bacterial membranes to reduce biofilm biomass and viability in Staphylococcus aureus and Pseudomonas aeruginosa models [266,267,268]. Chitosan- or quaternary-ammonium-coated MSNs decrease minimum biofilm eradication concentration (MBEC) values through synergistic interactions with antibiotics [269].

Antiviral efficacy: Silica nanocarriers have shown antiviral action through surface adsorption or delivery to prevent viral entry or viral replication [270]. The virucidal activity of silver/copper-doped MSNs has been seen against enveloped viruses. SARS-CoV-2, herpes simplex virus (HSV), and influenza A are inhibited by the lipid envelope disruptor and oxidative stress-inducer [271,272,273]. MSNs functionalized with antiviral drugs (e.g., oseltamivir, acyclovir) enhance the uptake of antiviral drugs into the mucosal cells and increase the stability of the drugs [274].

Antiparasitic effect: Natural compounds or metal ions (Ag, Zn, Cu) incorporated in hybrid MSNs exhibit anti-leishmanial and anti-malarial properties that inhibit the parasite’s metabolism and induce damage through ROS-dependent pathways [275,276,277,278]. Green-synthesized MSNs using the plant polyphenols or chitosan further enhance additional selectivity against less cytotoxic parasites in mammalian cells [279].

**Figure 6:**
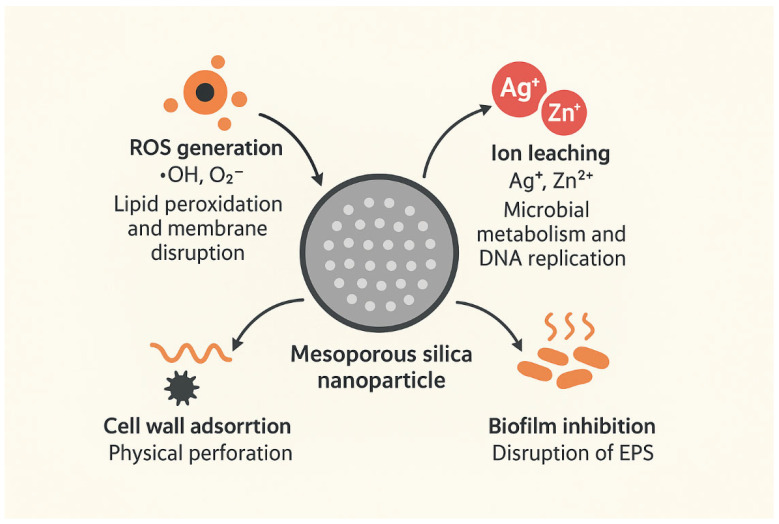
Antimicrobial mechanisms of mesoporous silica nanoparticles (MSNs), showing reactive oxygen species generation, ion release, cell wall adsorption and disruption, and inhibition of biofilm formation leading to microbial inactivation.

Collectively, these antiparasitic and antimicrobial MSN-based systems demonstrate the versatility of MSNs as multifunctional nanoplatforms capable of addressing microbial resistance, chronic infections, and neglected tropical diseases [265,280,281]. Shown in Figure 6 is the antimicrobial activity of mesoporous silica nanoparticles (MSNs).

### 6.7. Overarching Challenges and Translation Considerations

Translational challenges for this set of applications are broadly common and include problems such as the following: (i) reproducible, scalable manufacturing with close control of size and porosity; (ii) through pharmacokinetic and ADME profiling, including degradation into orthosilicic acid and assessment of organ retention; (iii) uniform immunotoxicity testing to analyze protein corona effects as well as unwanted immune stimulation; and (iv) regulatory path definition for complex hybrid and multi-component materials. All these issues need to be addressed by standardized assays, GLP-grade chronic toxicity testing, and large-scale green production in order to push MSN platforms towards clinical trials. Although MSNs have demonstrated high efficacy in preclinical disease models, translation to humans remains slow. Inconsistent animal models, incomplete chronic toxicity data, and a lack of harmonized pharmacological endpoints continue to impede regulatory acceptance.

## 7. Biocompatibility, Safety, and In Vivo Behavior

The biocompatibility and biosafety of mesoporous silica nanoparticles (MSNs) and hybrid derivatives are particularly important in assessing the translational potential of MSNs in biomedical applications. Although MSNs are found to be generally safe for administration by the U.S. FDA when they degrade to silicic acid, the behavior of MSNs in biological systems is multifaceted and involves size, surface charge, porosity, coating chemistry, and dosage [222,282]. It is therefore important to know the cytotoxicity, hemocompatibility, immunogenicity, biodistribution, and long-term degradation in order to optimize MSN design for in vivo use.

### 7.1. Cytotoxicity and Hemocompatibility

Cytotoxicity tests have shown that unmodified MSNs, particularly those with high surface reactivity or residual surfactant content, can induce oxidative stress, membrane damage, and mitochondrial dysfunction at higher concentrations (>200 μg/mL) [283]. Surface modification with polymer coatings such as PEG, chitosan, or phospholipids does considerably reduce nonspecific protein adsorption and improve biocompatibility [64].

Hemocompatibility tests indicate that well-functionalized MSNs cause negligible hemolysis (<5%) and maintain the shape of erythrocytes under physiological conditions [284]. Cationic surface moieties, although promoting cell uptake, can lead to hemolysis or platelet activation; hence, charge neutrality or zwitterionic coatings are advisable for systemic delivery [285]. Some recent in vitro research has revealed that hybrid MSNs with polymeric or lipid shells exhibit better hemocompatibility than bare silica, following ISO 10993 standards for biomaterials [286,287]. In terms of clinical safety, mesoporous silica nanoparticles (MSNs) have demonstrated generally favorable biocompatibility profiles in both oral and injectable formulations. For oral administration, in vivo studies indicate that MSNs are largely non-toxic at doses below 200 mg/kg, with gradual biodegradation to orthosilicic acid (Si(OH)_4_), a naturally excretable metabolite [97,288]. Their high surface area facilitates drug absorption without inducing gastrointestinal irritation or systemic toxicity. For injectable routes, intravenous and intratumoral administration of functionalized MSNs have shown minimal hemolysis, limited complement activation, and negligible acute organ toxicity at therapeutic concentrations (<50 mg/kg) [289,290]. The use of surface coatings such as PEG, chitosan, or phospholipids further improves blood compatibility and reduces immunogenicity [291]. Nonetheless, long-term accumulation, chronic exposure effects, and interspecies variation in clearance kinetics warrant continued investigation prior to clinical translation. Collectively, current evidence supports the safety of well-functionalized MSNs for both oral and parenteral use, highlighting their strong potential for clinical drug delivery applications [292].

### 7.2. Immunogenicity and Inflammatory Response

MSNs engage immune cells through endocytosis by dendritic cells and macrophages. Particle size and roughness affect cytokine secretion profiles and immune activation. For example, particles of lesser size (<100 nm) tend to elicit weaker proinflammatory responses compared to larger aggregates [293]. Surface PEGylation, biomimetic membrane coating, or lipid hybridization reduces complement activation and macrophage clearance and leads to extended systemic circulation [24].

Although most research documents low immunogenicity for properly passivated MSNs, some amine- or quaternary ammonium-modified surfaces have been known to upregulate TNF-α, IL-1β, and IL-6 release, proposing a potential for immunostimulation [294]. These effects need to be balanced based on application immunotherapy to take advantage of controlled immune activation, while drug delivery needs immune evasion.

### 7.3. Biodistribution, Clearance, and Degradation

Following systemic administration, MSNs are distributed mainly to the liver, spleen, and lungs through the mononuclear phagocyte system (MPS). Particle size significantly affects biodistribution: smaller MSNs (<50 nm) are able to escape hurried sequestration and exhibit renal excretion, while larger (>150 nm) accumulate in the liver and spleen [295]. Degradation is through the hydrolysis of Si–O–Si bonds to soluble orthosilicic acid (Si (OH)_4_), which is naturally eliminated in urine [296].

Hybrid MSNs with biodegradable polymers (such as poly (lactic-co-glycolic acid), polycaprolactone) exhibit enhanced clearance kinetics and decreased long-term retention [297]. Chronic exposure studies validate minimal accumulation and no histopathological abnormalities in principal organs at therapeutic doses (<50 mg/kg) [222]. Nevertheless, non-biodegradable inorganic hybrids can be longer-lasting and need careful long-term biokinetic surveillance.

### 7.4. Protein Corona Effects

When exposed to biological fluids, MSNs instantly adsorb serum proteins to create a protein corona, which determines their biological identity, cellular uptake, and immune detection [15]. This corona composition is influenced by nanoparticle size, surface chemistry, and charge. Hydrophobic or charged surfaces bind to opsonins (e.g., immunoglobulins, complement factors), enhancing clearance, whereas PEGylated or zwitterionic surfaces bind dysopsonins (albumin, clusterin), extending circulation [93].

Higher-order proteomic characterization (LC–MS/MS) has defined silica- and hybrid nanocarrier-specific corona signatures, implying that stealth or targeting behavior can be controlled through controlled corona generation [19]. Nevertheless, batch-to-batch variation in corona composition continues to be a key challenge for reliable pharmacokinetics and biological response.

### 7.5. Long-Term Fate and In Vivo Toxicology

Sub-chronic and chronic exposure in rodents and non-human primates demonstrates that biodegradable silica transforms to orthosilicic acid, which is excreted through renal pathways in a safe manner [291]. Long-term histological evaluation shows negligible inflammatory infiltrates and normal hepatic and renal function after repeat dosing. High-dose or uncoated silica transiently accumulates in macrophage-rich tissues, highlighting the requirement for thorough sub-chronic and chronic toxicity assessments [298].

In vivo imaging (MRI, PET) and inductively coupled plasma mass spectrometry (ICP–MS) are now routine tools for tracking the biodistribution and clearance kinetics of MSNs in real time. Future translational studies must incorporate pharmacokinetic modeling as a whole, genotoxicity, and reproductive toxicity testing to establish a general safety profile [299].

## 8. Scale-Up, Manufacturing, and Regulatory Considerations

Although much progress has been made in laboratory-scale synthesis, industrial application of MSNs is hampered by reproducibility, scalability, and regulation standardization. Manufacturing processes need to provide batch-to-batch uniformity in particle size, porosity, and surface chemistry while retaining biocompatibility and therapeutic efficacy.

### 8.1. Reproducibility and Batch Consistency

MSN synthesis via the sol–gel or templating paths is highly susceptible to conditions such as pH, temperature, surfactant concentration, and stirring rate. Small differences are able to alter pore morphology and drug capacity [24]. With recent developments in continuous-flow and microfluidic-assisted synthesis, reproducibility has been enhanced with uniform particle distribution achieved on gram-to-kilogram scales [213]. Automation and real-time monitoring of the process by in-line spectroscopy and dynamic light scattering are novel instruments to maintain reproducibility under good manufacturing practice (GMP) conditions [300].

### 8.2. Scalability and Process Optimization

For production on the clinical scale, batch processes would be replaced by continuous or semi-continuous reactors that would permit the fine control of reactant mixing and residence time. Surfactant removal and surface functionalization processes would require environment-friendly and cost-effective alternatives to toxic solvents. “Green synthesis” involving biotemplates, ionic liquids, or supercritical fluids is becoming popular for MSN production in a sustainable way [104].

Post-synthetic elaborations like polymer grafting or ligand conjugation need also to be standardized in order to reach equivalent levels of functionalization, which in turn affect pharmacokinetic properties and therapeutic activity.

### 8.3. Stability, Storage, and Shelf Life

MSN formulations are stable in dry powder form but may undergo aggregation and hydrolysis in aqueous dispersions. Lyophilization with cryoprotectants (e.g., trehalose, mannitol) preserves colloidal stability when stored and reconstituted [301]. Hybrid nanoparticles incorporating lipids or polymers require cold-chain storage to ensure coating integrity and prevent oxidation. Shelf-life according to ICH stability guidelines ensures approval [300].

### 8.4. Regulatory and Quality Control Perspective

Regulatory qualification of MSN-based nanomedicines must meet international standards (ISO 10993, ICH Q8–Q10) [287,302,303,304]. The challenges are establishing nanomaterial characterization standards (size distribution, surface area, zeta potential) and defining acceptable residual surfactant or solvent levels [305].

The FDA and EMA recommend case-by-case evaluation for nanocarriers, emphasizing toxicity, immunogenicity, pharmacokinetics, and stability data. Few silica-based materials (e.g., Cornell dots) have reached the stage of clinical trials in imaging and drug delivery so far, underscoring the need for harmonized regulation [306]. Sophisticated modeling, artificial intelligence-based quality control, and high-throughput screening equipment are under development to aid in expedited safety testing and product launch [24].

## 9. Challenges, Limitations, and Future Outlook

Despite the high promise of MSNs and hybrid systems in nanomedicine, various scientific and translational hurdles must be overcome to make clinical use successful.

### 9.1. Current Limitations

Significant limitations include nanoparticle aggregation in in vivo conditions, premature release of the drug, and residual non-degraded structures in vivo [307]. Interactions with biomedicine alter surface characteristics, resulting in uneven biodistribution and immune response. Further, the control of multifunctional architectures (e.g., co-delivery or theragnostic hybrids) without sacrificing reproducibility remains problematic. Toxicological variability between in vivo models and the lack of standard protocols for testing complicate translation to the clinic [24].

### 9.2. Smart and Programmable Silica Hybrids

Next-generation MSNs are engineered as “programmable nanocarriers” that can react to multi-stimuli, modulate drug release with feedback, and provide real-time imaging. Their capability to incorporate stimuli-sensitive polymers, DNA-based nano-valves, or biorthogonal click linkers allows for the precise temporal control of therapeutic intervention [308]. However, the use of DNA-based components may raise concerns regarding in vivo immunogenicity and recognition by Toll-like receptors (TLR9), necessitating sequence optimization and chemical modification (e.g., CpG depletion, methylation) to minimize innate immune activation [309]. Integration with extrinsic stimuli such as magnetic fields, ultrasound, or NIR light offers additional opportunities for non-invasive activation at targeted sites. These multi-systems are promising in precision oncology, neurodegenerative disease treatment, and regenerative medicine [310].

### 9.3. Integration with Emerging Modalities

Hybrid MSNs are being engineered to synergize with cell therapy, immunotherapy, and gene editing platforms. For example, MSN scaffolds are employed to boost CAR-T cell proliferation, local cytokine release, or CRISPR/Cas9 delivery efficiency [248]. Their hybridization with biomimetic membranes or exosomes can enhance biocompatibility and homing capability, integrating nanotechnology with cell-based therapeutics [311].

### 9.4. Sustainable and Green Synthesis

Environmentally friendly, energy-efficient, solvent-free synthetic paths are crucial for massive production and environmental protection. Biogenic templating, enzymatic catalysis, and plant extract-based silica synthesis are new sustainable pathways with minimal harmful waste and expense [312]. Green synthesis also facilitates increased regulatory acceptance by concordance with sustainable development goals (SDG 12: Responsible Consumption and Production).

### 9.5. Path Forward for Clinical Translation

For MSNs to move from the laboratory to the clinic as clinically accepted therapeutics, collaborative interactions among materials scientists, pharmacologists, toxicologists, and regulatory agencies are crucial. The development of standardized characterization methods, proven toxicity assays, and predictive computational models will make risk assessment more efficient. Reproducibility, in vivo correlation between efficacy and safety, and GMP manufacturing that is cost-efficient should be prioritized [300].

**Figure 7:**
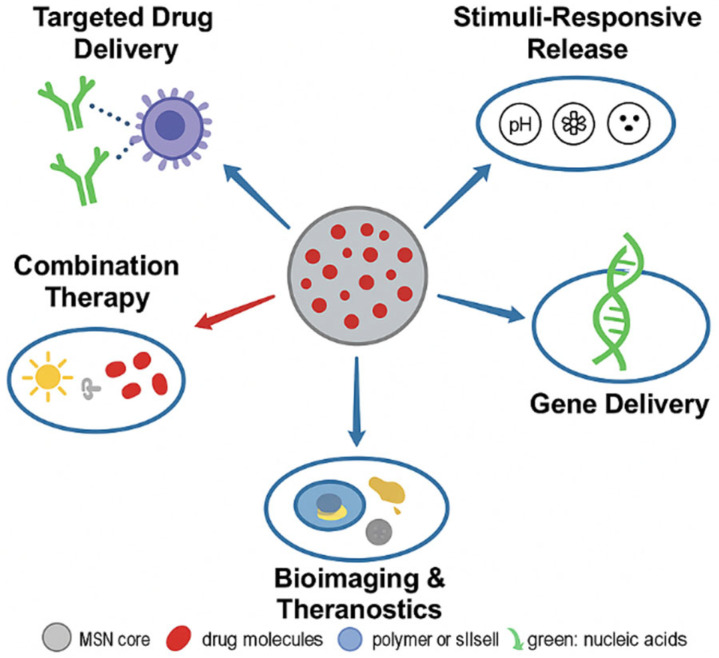
Advanced pharmaceutical applications of mesoporous silica nanoparticles (MSNs). Schematic representation showing MSNs used for targeted drug delivery, stimuli-responsive release, gene delivery, bioimaging and theranostics, and combination therapy. Dotted lines indicate pathways or interactions that are conditional, indirect, or stimuli-dependent, illustrating the routes by which MSNs respond to specific triggers in the biological environment.

In summary, mesoporous silica and hybrid nanocarriers represent one of the most mature inorganic platforms in nanomedicine, yet their clinical translation remains constrained by issues of reproducibility, biodegradation kinetics, and immunogenicity. Among current directions, biomimetic hybrids and DNA-programmed molecular valves appear most promising for achieving programmable, patient-tailored therapies. Moving forward, the field must prioritize standardized in vivo pharmacokinetic modeling, chronic toxicity profiling, and scalable, green synthesis pipelines aligned with regulatory frameworks. Only through such integrative approaches can MSN technology progress from descriptive research to clinical reality. The multifunctional design and translational potential of MSNs are summarized schematically in Figure 7. Recent research assessing the cytotoxicity and biodegradability of MSNs is presented in Table 4 and reflects surface functionalization’s impact on safety profiles.

## 10. Conclusions

Mesoporous silica nanoparticles (MSNs) and their hybrid derivatives represent a rapidly developing class of nanocarriers with exceptional drug delivery and biomedical applications. Their unique physicochemical properties like high surface area, tunable pore size, regulable morphology, and facile surface modification enable efficient drug loading, targeted delivery, and the controlled release of therapeutic drugs. These properties position MSNs as leading contenders for developing next-generation nanomedicines that can increase efficacy, restrict systemic toxicity, and provide precision therapy.

Over the past few years, there have been tremendous advances in MSN design and functionalization, particularly through the incorporation of polymers, lipids, biomolecules, and inorganic moieties. These hybrid and stimulus-responsive platforms have enabled site-specific, on-demand release triggered by environmental stimuli such as pH, redox gradients, or enzyme reactions. These advances have prolonged the biomedical applications of MSNs, including chemotherapy for cancer, gene and nucleic acid delivery, antimicrobial therapy, vaccine formulation, and imaging diagnostics. Collectively, these studies demonstrate the potential of MSNs as multifunctional platforms for therapeutic and theragnostic pursuits.

Although such promising progress was achieved, there are yet some scientific and translational challenges to be overcome. Challenges of large-scale synthesis, reproducibility issues, long-term biodegradability, immune interactions, and regulatory approval need to be addressed systemically. Uniform characterization protocol, biosafety testing, and quality control protocols will have to be developed to guarantee uniform performance and clinic-readiness. Harmonization of international regulatory guidelines for nanomedicine will also be necessary to facilitate clinical trials and the commercialization of therapeutics using MSN.

In the forthcoming future, research will have to be centered on intelligent, programable MSN architectures, green and sustainable routes of synthesis, and hybrid integrative systems with the capability for the integration of drug delivery with imaging, immunomodulation, or gene therapy. Interdisciplinary collaborative research by material scientists, pharmacologists, toxicologists, and clinicians will be critical in overcoming the current challenges and facilitating clinical translation. With persistent innovation and strict safety verification, mesoporous silica and hybrid nanoparticles promise to transform targeted and individualized drug delivery in the next decade.

## Figures and Tables

**Table 1:** Overview of the main synthesis approaches for mesoporous silica and hybrid nanoparticles. Methods differ based on their precursor systems, templating methods, pore diameters, and functional advantages accessible for drug delivery and biomedical applications.

Method	Precursors/Reagents	Template/Surfactant Used	Typical Pore Size (nm)	Key Parameters/Conditions	Major Advantages	References
Sol–gel process	Tetraethyl orthosilicate (TEOS), tetramethyl orthosilicate (TMOS), ethanol, water, acid/base catalyst	Cetyltrimethylammonium bromide (CTAB), Pluronic P123	2–10	Hydrolysis condensation at mild temperature and controlled pH	Simple, reproducible, tunable pore size, scalable synthesis	[2,7,49]
Soft-templating	Silica precursors (TEOS/TMOS), polymeric or ionic surfactants	CTAB, F127, PEG	2–20	Surfactant–silica self-assembly under mild conditions	Excellent control over morphology and mesostructure	[50,51]
Hard-templating (nanocasting)	Silica precursors + solid template (carbon spheres, polystyrene, SBA-15)	Carbon/polymer templates	5–30	Template impregnation → calcination/etching	High structural precision, ordered pore network	[52,53]
Co-condensation/Grafting	TEOS + functional silanes (e.g., APTES, MPTMS)	CTAB/P123	2–8	Functional silanes added during or after synthesis	Enables in situ surface functionalization; stable covalent bonding	[54,55]
Hybridization (Polymer/Lipid/Metal composites)	Silica precursors + organic or metal components	Surfactant/lipid stabilizers	5–50	Sol–gel or microemulsion route with polymeric coating/metal doping	Enhanced biocompatibility, stability, and stimuli responsiveness	[13,56,57]
Green synthesis	Silica precursors + plant extracts/biomolecules	Biomolecule-assisted self-assembly	5–15	Conducted under mild, eco-friendly conditions	Non-toxic, sustainable, environmentally benign synthesis	[58,59]

**Table 2:** Functionalization and stimuli-responsive strategies of mesoporous silica and hybrid nanoparticles.

Functionalization Strategy	Stimuli Type/Trigger	Representative Drug/Model System	Mechanism/Outcome	References
Surface Grafting of Targeting Ligands	Passive (ligand–receptor interaction)	Doxorubicin-loaded folic acid-functionalized MSNs	Targeted delivery via receptor-mediated endocytosis; enhanced uptake in folate-receptor-positive cancer cells	[2,49,50]
PEGylation (Polyethylene Glycol Coating)	Passive (stealth modification)	Curcumin-loaded PEG–MSNs	Improves systemic circulation, reduces immune clearance, and enhances stability	[183,184,185]
pH-Responsive Polymer Coating (e.g., Polyacrylic acid, Chitosan)	Internal (acidic tumor microenvironment)	Doxorubicin, Cisplatin	pH-triggered swelling or degradation causes drug release in tumor environment	[53,54,186]
Redox-Responsive Functionalization (Disulfide Bridges, Thiol–PEG)	Internal (glutathione-mediated reduction)	Paclitaxel, Doxorubicin	Drug release triggered by intracellular redox potential; selective cytotoxicity to cancer cells	[55,187,188]
Enzyme-Responsive Gatekeepers (Peptide, Gelatin, or Hyaluronic acid coating)	Internal (MMPs, hyaluronidase activity)	Doxorubicin, Gemcitabine	Enzyme-mediated cleavage removes caps, releasing the payload locally	[189,190,191,192]
Light-Triggered Release Systems (Photothermal/Photocleavable groups)	External (NIR/UV light irradiation)	Camptothecin, Doxorubicin	Light-induced heating or bond cleavage triggers controlled release	[193,194,195]
Temperature-Responsive Polymers (e.g., PNIPAM, Pluronic F127)	External (temperature variation)	5-Fluorouracil, Ibuprofen	Polymer phase transition near body temperature controls drug release rate	[75,196,197]
Magnetic/Redox Hybrid Nanoparticles	Combined (magnetic field + redox potential)	Doxorubicin + Fe_3_O_4_@MSN	Dual-triggered release with magnetic targeting and intracellular reduction	[198,199]
Gatekeeper-Based Systems (β-cyclodextrin, Mesopore Caps, Quantum Dots)	Multiple (pH, redox, light)	Doxorubicin, Rhodamine B	Controlled, multi-stimuli-responsive release via pore opening	[183,200,201]
Co-delivery Systems (Drug + siRNA/Drug + Imaging agent)	Multiple (pH/redox/enzyme)	Doxorubicin + siRNA, Cisplatin + Quantum dots	Enables combination therapy and real-time tracking; improved efficacy	[183,202,203]

**Table 3:** Recent biomedical applications of mesoporous silica and hybrid nanoparticles, summarizing carrier types, target diseases, model drugs, and therapeutic outcomes, highlighting advances in targeted, stimuli-responsive, and multifunctional nanocarrier systems.

Type of MSN/Hybrid System	Target Disease/Application Area	Loaded Drug or Cargo	Key Outcome/Findings	References
Folic acid-functionalized MSNs (FA-MSNs)	Breast cancer	Doxorubicin (DOX)	Enhanced selective uptake in MCF-7 cells; pH-responsive release and reduced off-target toxicity.	[49,50,237]
Chitosan-coated MSNs (CS-MSNs)	Colon cancer	5-Fluorouracil (5-FU)	Improved intestinal stability, delayed release in acidic pH, and higher cytotoxicity to HT-29 cells.	[168,183,184]
Magnetic hybrid MSNs (Fe_3_O_4_@MSNs)	Liver cancer	Doxorubicin	Dual magnetic targeting and redox-triggered release; increased intracellular accumulation in HepG2 cells.	[55,198,199]
PEGylated mesoporous silica nanocarriers	Glioblastoma multiforme	Temozolomide (TMZ)	Extended circulation time and improved blood–brain barrier permeability for CNS drug delivery.	[185,238]
Hyaluronic acid (HA)-modified MSNs	Lung cancer	Cisplatin	Enhanced CD44 receptor-mediated targeting and reduced systemic toxicity in A549 cells.	[239,240]
Lipid–silica hybrid nanoparticles (LSHNs)	Pancreatic cancer	Gemcitabine	Improved drug encapsulation efficiency, controlled release, and higher apoptotic activity in PANC-1 cells.	[53,241]
pH/redox dual-responsive MSNs	Ovarian cancer	Paclitaxel	Controlled release under acidic and reductive conditions; selective cytotoxicity in SKOV-3 cells.	[242,243]
Enzyme-responsive peptide-capped MSNs	Prostate cancer	Curcumin	MMP-triggered gatekeeper removal led to enhanced local release and reduced tumor growth.	[189,244]
MSNs loaded with antibacterial agents (Ag^+^, vancomycin)	Bacterial infections	Silver ions, Vancomycin	Sustained antibacterial activity against *S. aureus* and *E. coli*; improved biofilm inhibition.	[245,246]
MSN-based vaccine nanocarriers	Immunotherapy/Vaccine delivery	Peptide antigens, CpG adjuvants	Stronger antigen presentation and immune activation; enhanced IgG and cytokine response in mice.	[247,248]
MSNs co-loaded with DOX and siRNA (theranostic)	Multidrug-resistant breast cancer	Doxorubicin + MDR1 siRNA	Synergistic gene–drug therapy; suppression of P-gp expression and increased cancer cell apoptosis.	[183,249]
Cerium oxide-doped silica nanohybrids (CeO_2_–MSNs)	Oxidative stress/Neuroprotection	Antioxidant enzymes	ROS scavenging activity and neuronal protection in oxidative injury models.	[250,251]
Bone-targeted MSNs (alendronate-functionalized)	Osteosarcoma/Bone regeneration	Doxorubicin, BMP-2	Controlled release and enhanced osteogenic differentiation with localized drug action.	[252,253,254]

**Table 4:** Biocompatibility and safety evaluation of mesoporous silica and hybrid nanoparticles, summarizing cytotoxicity, biodegradation, and immune responses, with surface functionalization improving stability, immune tolerance, and clinical potential.

Nanoparticle System	Experimental Model	Cytotoxicity/In Vitro Findings	In Vivo/Biodegradation Outcomes	Immune or Hemocompatibility Response	References
Bare MSNs (50–100 nm)	Human fibroblasts (HFF-1), HeLa cells	Dose-dependent cytotoxicity above 200 µg/mL; low ROS generation at therapeutic concentrations.	Partial biodegradation in lysosomal conditions within 7–10 days; renal clearance confirmed.	No significant inflammatory cytokine elevation (IL-6, TNF-α) at ≤100 µg/mL.	[10,21,213]
Amino-functionalized MSNs (NH_2_–MSNs)	HepG2, MCF-7 cells	Enhanced cell viability (>85%) up to 150 µg/mL; improved dispersibility.	Moderate degradation observed via Si release in serum (24–72 h).	Slight complement activation; no hemolytic activity at physiological pH.	[3,49,313]
PEGylated MSNs (PEG–MSNs)	Caco-2, RAW 264.7 cells	PEGylation reduced cellular uptake but improved long-term biocompatibility.	Prolonged blood circulation and minimal liver accumulation in mice.	Negligible macrophage activation; improved stealth behavior in vivo.	[314,315,316]
Chitosan-coated MSNs (CS–MSNs)	HT-29, A549 cells	Non-toxic up to 250 µg/mL; enhanced mucoadhesion and cellular internalization.	Gradual degradation with complete clearance after 30 days in BALB/c mice.	Mild immune stimulation beneficial for oral vaccine adjuvant applications.	[317,318,319]
Lipid–silica hybrid nanoparticles (LSHNs)	MDA-MB-231, HEK-293 cells	High viability (>90%) and reduced oxidative stress; favorable drug encapsulation.	Stable in plasma; enzymatic lipid degradation followed by silica dissolution.	No hemolysis or complement activation detected in vitro.	[320,321]
Magnetic Fe_3_O_4_@MSNs	PC-3, HepG2 cells	Minimal cytotoxicity below 100 µg/mL; increased internalization via endocytosis.	Accumulation in reticuloendothelial organs (liver, spleen) reduced by PEG coating; excreted via biliary route.	No abnormal hematological or immunological response in murine models.	[322,323,324]
pH/Redox dual-responsive hybrid MSNs	SKOV-3, MCF-7 cells	Excellent cytocompatibility and selective toxicity in acidic/redox microenvironments.	Efficient in vivo degradation through glutathione-mediated silica dissolution.	Immune-neutral profile; no cytokine elevation in serum.	[325,326]
Enzyme-responsive peptide-capped MSNs	LNCaP cells, macrophage assays	Safe at therapeutic concentrations; enzymatic cleavage did not trigger inflammation.	Rapid clearance after enzymatic degradation; minimal residual silica in major organs.	Reduced macrophage uptake and cytokine secretion compared to uncoated MSNs.	[244,326,327]
Alendronate-functionalized MSNs (Bone-targeted)	MG-63 osteoblasts, rat femur model	Non-cytotoxic and osteoinductive; promoted cell proliferation and ALP activity.	Gradual silica resorption observed in bone microenvironment.	No systemic immune or inflammatory response detected.	[328,329]
MSNs co-loaded with drug/imaging agents (Theranostic hybrids)	4T1 tumor-bearing mice	Dual-functional MSNs exhibited low systemic toxicity; stable in serum.	Efficient biodegradation after 2–3 weeks; visualized via MRI tracking.	No hematological abnormalities or organ damage observed histologically.	[330,331,332]

## Data Availability

The data presented in this study are available within the article.
